# Special Issue “Reproductive Health Concerns for Women”

**DOI:** 10.3390/life11111274

**Published:** 2021-11-21

**Authors:** Szu-Ting Yang, Wen-Ling Lee, Peng-Hui Wang

**Affiliations:** 1 Department of Obstetrics and Gynecology, Taipei Veterans General Hospital, Taipei 112, Taiwan; ss19910224@hotmail.com; 2 Institute of Clinical Medicine, National Yang-Ming University, Taipei 112, Taiwan; 3 Department of Obstetrics and Gynecology, National Yang Ming Chiao Tung University, Taipei 112, Taiwan; 4 Department of Medicine, Cheng-Hsin General Hospital, Taipei 112, Taiwan; 5 Department of Nursing, Oriental Institute of Technology, New Taipei City 220, Taiwan; 6 Female Cancer Foundation, Taipei 104, Taiwan; 7 Department of Medical Research, China Medical University Hospital, Taichung 404, Taiwan

There are specific health issues concerning the reproductive age of women, including inflammatory disease, fertility, and childbearing. Therefore, it is important to focus our attention on female health, as it is related to the impairment of reproductive functions in women. 

Montero-Vilchez et al. investigated the characteristics of hidradenitis suppurativa during childbearing age [1]. This disease attacks younger women with unfulfilled reproductive desires. It is important for clinicians to consider reproductive desires when treating these women. Hidradenitis suppurativa is an inflammatory disease which not only has a great impact on patient’s quality of life, but also significantly impairs the fertility. Based on this consideration, the authors in the same group further provide the rationale and clinical evidence regarding the treatment for hidradenitis suppurativa [2]. Generally, treatment options for hidradenitis suppurativa include weight reduction, smoking cessation, topical clindamycin, oral contraceptive pills, oral retinoids, immunosuppressants and surgical management [3]. Montero-Vilchez’s study revealed that oral contraceptive pills are effective against hidradenitis suppurativa, especially in individuals whose symptoms worsen during the menstrual period [2]. Although the current article is promising, we still know little regarding the effects and exact pathophysiology of the hormonal impact on the development and management of hidradenitis suppurativa [3].

Besides female hormone-related skin disorders as shown above [1,2], leiomyoma of the uterus may be one of the most common benign disorders in women at reproductive age [4]. Among the various kinds of classification of leiomyomas, submucosal myoma is often associated with symptoms and health problems, which not only results in heavy menstruation and subsequent anemia, but may also impair the fecundity of women, increasing the risk of infertility and pregnancy loss [5]. With advanced technology and the improvement of patient care, minimally invasive procedures have become much more attractive and popular in modern clinical practice [5]. The hysteroscopic approach to perform myomectomy is a fast, relatively non-invasive, but effective surgical method for the management of submucosal myoma, although some uncertainties are still debated [5,6]. For example, intrauterine adhesion (IUA) after hysteroscopic myomectomy is the biggest challenge and raises great concern owing to the significant impairment of reproductive functions and abnormal uterine bleeding [6]. Therefore, we are happy to investigate articles focusing on this issue. Dr. Huang and her colleagues attempted to evaluate the efficacy of using hyaluronic acid (HA) gels after hysteroscopic myomectomy to prevent IUA development and formation [7]. The results confirm a constantly and relatively high risk of varied degrees of IUA formation after hysteroscopic myomectomy (21.4%). Their study also identifies the encouragement of routine application of HA gels as a prevention strategy after hysteroscopic myomectomy [7]. Women treated with HA gels have a statistically significant lower incidence of IUAs than non-treated women (13% vs. 39%), and additionally, women receiving the HA gel treatment have a significantly reduced severity of IUA than women in the no-HA treatment group [7]. In the current special issue, based on the positive benefits of application of HA as a preventive strategy for patients after hysteroscopic myomectomy [7], a systematic review and meta-analysis was conducted to investigate the effect of HA on the primary prevention of IUA formation after hysteroscopic myomectomy [8]. A pooled analysis of three randomized controlled trials shows a statistically significant reduction in the development of IUA postoperatively, with an odd ratio (OR) of 0.29, and a 95% confidence interval (CI) of 0.12~0.70, suggesting that the use of HA gel in patients after hysteroscopic myomectomy can significantly reduce de novo IUA [8]. Although this meta-analysis was limited by a small sample size, it is still a remarkable study to provide us with a reference to take HA gels into consideration to prevent IUA.

In addition to the aforementioned hormone-related diseases published in this Special Issue, the studies addressing the enhancement of fecundity are also included. The current assisted reproductive technique (ART) involving oocyte retrieval and the subsequent in vitro fertilization, or intracytoplasmic sperm injection and embryo transfer (IVF/ICSI-ET), has been widely used for the therapy in a certain population of couples with infertility [9,10]. However, advanced maternal age (AMA) may be one of the biggest challenges during the ART due in part to diminished ovarian reservation, poor oocyte quality, chromosome aneuploid, and a lower implantation rate [11]. Based on the concept of the increased oxidative stress and blunted antioxidant signaling in aged ovary, Katz-Jaffe et al. utilized a supplement containing a natural antioxidant agent to show how upregulated β-adrenergic signaling, downregulated apoptosis, and proinflammatory signaling variably affected cell growth and antioxidant pathways as well as the increased expression of antioxidant genes, such as *GPX1*, *SOD2*, and *GSR* in the aged oocyte [11]. In clinical practice, using antioxidant supplements prior to the IVF cycle in women older than 39 also showed a promising result for AMA patients [11], suggesting an understanding of the ageing process of the ovary and that a strategy of using various kinds of agents to slow down the ageing process of the ovary may be possible, although there is still a long way to go [12,13]. As a prerequisite of comparable reproductive outcomes, it is crucial to design a more patient-friendly ovarian stimulation protocol. Huang et al. evaluated the efficacy of progestin-primed ovarian stimulation protocol (PPOS) during ART cycles [14]. The authors found that using the PPOS protocol for patients provides the following benefits, such as less visits and less needle injection compared to using conventional ovarian stimulation protocols. Remarkably, and impressively, women using PPOS protocol with oral medroxyprogesterone acetate showed competent stimulation and reproductive outcomes, suggesting that this friendly PPOS is not inferior to the conventional ovarian stimulation protocols. Further randomized controlled trials are needed to provide more evidence, in addition to a further study to identify individuals that benefit more. After the improvement of the quality and quantity of oocyte, the following IVF/ICSI-ET becomes much more critical, since implantation failure is also a key step during the ART, resulting in the urgent need to determine the key factor influencing embryo implantation [15]. Lv et al. analyzed single-cell sequencing data and further explored the transcriptomic changes in endometrial stromal cells after coculturing with trophoblast cells to show 1783 upregulated genes and 569 down regulated genes in the cocultured embryos [16]. Additionally, the weight gene co-expression network and gene ontology analysis of these differentially expressed genes (DEG) showed a higher instance of *RAMP1*, *LTBP1*, and *LRP1* in endometrial stromal cells after coculture, indicating the enrichment of biological processes in blood vessel development and female pregnancy [16]. 

When women conceive, there are numerous challenges during pregnancy. Some diseases occur before conception, during pregnancy, and in the postpartum period. Disease control before conception is always recommended. However, sometimes, it is hard to achieve, since some are exacerbated during pregnancy. Therefore, appropriate management is essential for a better pregnancy outcome. Goker-Alpan et al. investigated pregnancy outcomes in women with late-onset Pompe disease [17]. Although obstetric or neonatal complications were not significantly increased in these pregnant women, the stillbirth rate was increased [17]. Hyperemesis gravidarum, characterized by persistent vomiting, weight loss of more than 5%, ketonuria, electrolyte abnormalities (hypokalemia), and dehydration (high urine specific gravity) is a severely uncomfortable disorder, resulting in the diminishment of the woman’s quality of life, a significant contribution to health care costs, and time lost from work, which can be associated with worse perinatal outcomes [18]. This usually occurs in the first trimester. Therefore, it is important to recognize the risk factors associated with hyperemesis gravidarum, since this early intervention and treatment may avoid the subsequent bad outcomes as discussed above. Kim’s group found that underweight or lower waist circumference, primiparity, multiple pregnancies, and bearing female babies are risk factors for the development of hyperemesis gravidarum [19]. Preeclampsia is one of the most notable pregnancy-related complications. Much research has aimed to find out sensitive and reliable markers to predict the risk and development of preeclampsia, and determine the severity of disease, although many issues still need to be overcome [20]. The outcome of early-onset is believed to be worse compared to that of late-onset. However, Kornacki’s group attempted to compare the difference of the endothelial injury between the early-onset and late-onset preeclampsia [21]. It is interesting to find that there is no statistically significant difference of degree for endothelial injury, using serum concentrations of hyaluronan and serum level of soluble vascular cell adhesion molecule-1 (sVCAM-1) as tools [21]. Finally, Vega et al. found that women with IgM antibodies against the phosphatidylserine-prothrombin complex (aPS/PT) titers having higher pulse wave velocity (PWV) levels [22]. Pulse wave velocity level is one of the cardiovascular parameters. Vasoconstriction indicates an increase of the PWV level. Both studies provide us with another perspective to evaluate preeclampsia, although further robust evidence is required.

In summary, this collection includes different points of view regarding the issue of reproductive health concerns for women, ranging from health care for an inflammatory disease and intrauterine adhesion, to ART considerations and diseases affecting pregnancy outcomes. These articles remind us of the importance of the special considerations of reproductive women. To provide the better care for women who need fertility preservation, and the fertility enhancement of women during their reproductive age, the 2nd edition of Special Issue “Reproductive Health Concerns for Women, 2.0” in *Life* is also announced. We are looking forward to seeing much valuable information and experience dissemination using this platform (https://www.mdpi.com/journal/life/special_issues/reprod_health_2). We welcome your submissions. 
